# Biomimetic Use of Food-Waste Sources of Calcium Carbonate and Phosphate for Sustainable Materials—A Review

**DOI:** 10.3390/ma17040843

**Published:** 2024-02-09

**Authors:** Sara Piras, Saniya Salathia, Alessandro Guzzini, Andrea Zovi, Stefan Jackson, Aleksei Smirnov, Cristiano Fragassa, Carlo Santulli

**Affiliations:** 1 School of Science and Technology, Chemistry Section, Università di Camerino, Via Madonna delle Carceri, 62032 Camerino, Italy; sara.piras@unicam.it (S.P.); alessandro.guzzini@unicam.it (A.G.); 2 School of Pharmacy, Università di Camerino, Via Sant’Agostino 1, 62032 Camerino, Italy; saniya.salathia@unicam.it (S.S.); andrea.zovi@unicam.it (A.Z.); stefan.jackson@unicam.it (S.J.); aleksei.smirnov@unicam.it (A.S.); 3 Department of Industrial Engineering, Alma Mater Studiorum Università di Bologna, 40133 Bologna, Italy; cristiano.fragassa@unibo.it; 4 School of Science and Technology, Geology Section, Università di Camerino, Via Gentile III da Varano 7, 62032 Camerino, Italy

**Keywords:** calcium carbonate, hydroxyapatite, mollusk shells, eggshells, snail shells, cuttlebone fish

## Abstract

Natural and renewable sources of calcium carbonate (CaCO_3_), also referred to as “biogenic” sources, are being increasingly investigated, as they are generated from a number of waste sources, in particular those from the food industry. The first and obvious application of biogenic calcium carbonate is in the production of cement, where CaCO_3_ represents the raw material for clinker. Overtime, other more added-value applications have been developed in the filling and modification of the properties of polymer composites, or in the development of biomaterials, where it is possible to transform calcium carbonate into calcium phosphate for the substitution of natural hydroxyapatite. In the majority of cases, the biological structure that is used for obtaining calcium carbonate is reduced to a powder, in which instance the granulometry distribution and the shape of the fragments represent a factor capable of influencing the effect of addition. As a result of this consideration, a number of studies also reflect on the specific characteristics of the different sources of the calcium carbonate obtained, while also referring to the species-dependent biological self-assembly process, which can be defined as a more “biomimetic” approach. In particular, a number of case studies are investigated in more depth, more specifically those involving snail shells, clam shells, mussel shells, oyster shells, eggshells, and cuttlefish bones.

## 1. Introduction

In the last decades, the attention paid to the use of calcium carbonate (CaCO_3_) as a reinforcing and hardening filler in composites, starting from cementitious composites then gradually extending to polymer ones, has increasingly emerged [1]. Calcium carbonate is obtainable from limestone quarries; however, the competition with its use in cement makes it gradually more difficult to respond to the demand for the mineral for other uses [2].

In the particular case of commodity polymers, such as polypropylene (PP) and poly (vinyl chloride) (PVC), the introduction of ceramic fillers, such as calcium carbonate, talc, feldspar, alumina, etc., has a particular significance for a number of reasons. These include improving surface finishing and controlling material rheology (and hence manufacturing) while increasing hardness and impact resistance [3]. This brings about the creation of composites with small aspect ratio (length/diameter) particles, which have also been extensively studied over the years, since particle size and interfacial adhesion have a significant effect on the strength, stiffness, and toughness of the material [4]. A typical substitute for mineral-extracted calcium carbonate is, for example, eggshell, which is significantly wasted in food industry, is able to provide resources for countless biotechnological applications and yet is also of interest in the construction industry [5]. In addition, the quantities provided do appear particularly striking and worthy of consideration: a China-limited analysis suggested that, in 2017, over 1 million tons of calcium carbonate were presumed to be available from eggshell waste, whilst a second noteworthy—though less pure—source of CaCO_3_ can be seashells obtained from aquaculture and the consumption of seafood [6].

**Figure 1:**
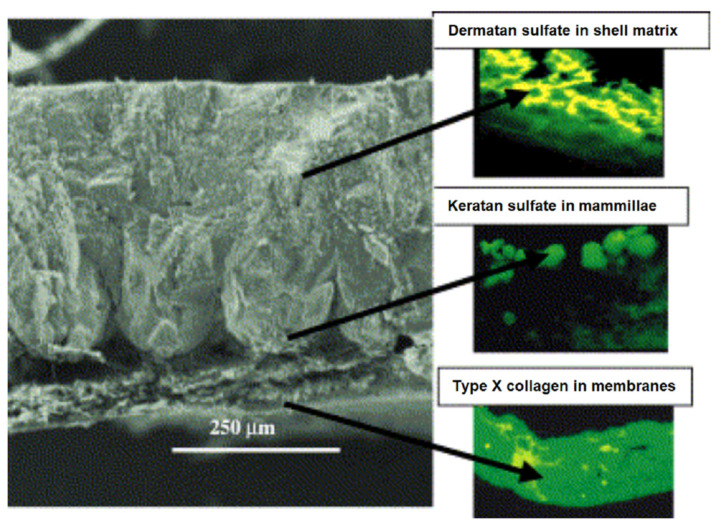
Macromolecules forming eggshells (left, 170× magnification) and how they are localized. Right (top to bottom). Immunofluorescence: 1. Dermatan sulfate in the shell matrix. 2. Keratan sulfate in the mammillae. 3. Type X collagen in the shell membranes (400× magnification) [7].

The bare substitution of calcium carbonate from mining activities with that obtained from biological sources, also referred to as “bio-calcium carbonate”, results in some gains in terms of resource depletion, yet it does not normally optimize use according to the specific characteristics of the single source of waste (granulometry, different polymorphs, e.g., calcite or aragonite, hierarchical microscopic patterns). Particular properties depend on the process of biomineralization, based on the development of macromolecules from dedicated specialized cells, such as in one of the simpler cases, namely chicken eggshell, as shown in Figure 1 [7].

In many cases, the use of biogenic calcium carbonate can be a bare chemical replacement for the extracted material, not accounting, therefore, for any specific or biomimetic characteristics regarding how biomineralization leads to specific self-assembly features in order to constitute the whole biological structure of the shell or exoskeleton [8]. Looking at the broader picture, the biogenic sources of calcium carbonate, depending on their mineralogical composition, tend to be more or less adapted to serve as an intermediary source for the synthesis of calcium phosphate, namely, to replace natural hydroxyapatite [9]. To achieve a complete transformation of calcium carbonate in hydroxyapatite, the specific stoichiometric Ca/P ratio, which is equal to 1.67, needs to be matched as closely as possible. For this reason, calcium carbonate and calcium phosphate are included in this review. The latter is produced by a calcination process at final temperatures normally in the region of 1000 °C, so as to obtain calcium oxide (CaO), which is subsequently subjected to a reaction with phosphoric acid (or phosphor-containing salt) to obtain synthetic hydroxyapatite.

**Figure 2:**
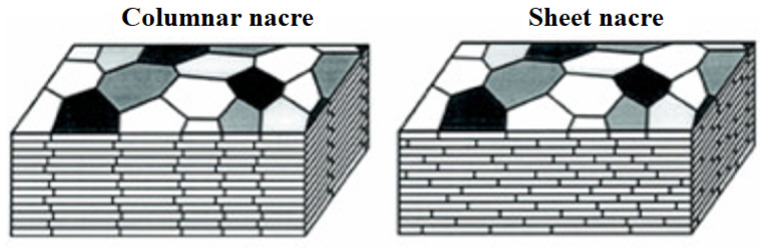
Two models of nacre structures (reported from [14]).

In practice, neglecting further considerations on its microstructural characteristics, bio-calcium carbonate may be suggested to have a solely mechanical role as a filler; however, it is essential during service, such as in preventing waviness by reducing the surface scratching of polyethylene [10]. This led, e.g., to the compilation of a specific review dealing with its effectiveness in terms of improving its tensile, flexural and impact strength [11]. On the other hand, knowing its microscopic structure and architecture can assist in highlighting how much the general performance of the composite can be improved by applying calcium carbonate from a specific source, as well as the extent of its repeatability. Some specific investigations into nacre are available: this is formed by a polymer/calcium carbonate layered structure which can be effectively mimicked by a composite laminate [12,13]. In practice, the most investigated mollusk shell from a biomimetic point of view is abalone, which has been so far of limited interest as a source of calcium carbonate; however, as a gastropod, it is in some ways suggestive of the usually much simpler structure of snail shells (other gastropods). Both gastropods and bivalves, like mollusks, such as clams, have an external shell structure, referred to as “periostracum”, which is followed by three distinct layers of calcium carbonate embedded in a very thin organic matrix. A recent study [14] further highlighted that, whatever the number of valves (one, as in the case of gastropods, or two, as in the case of bivalves) the basic “brick-and-mortar” nacre structure can be represented according to two possible models. The former has a layout based on separate columns (defined as “columnar”) and the latter on independent layers (defined as “sheet”), as depicted in Figure 2.

This review considered the large number of biological sources for calcium carbonate and for synthetic hydroxyapatite, destined, in the first case, for the production of composites and, in the second case, to biomaterials, hence for prospective biomedical applications. We decided investigate only a few particularly significant cases, originating from animals of interest from the food sector, namely from species living in a terrestrial environment, such as snail shells and eggshells, and others from species settled in a marine context, such as some types of seashells (clam, oyster and mussel) and bones from cuttlefish. Specific attention will be paid to the morphology of the filler and its effect on the polymer characteristics, both on the matrices offering expectations of success in composites, and on the specific processes aimed at the production of hydroxyapatite.

## 2. Biological (Biogenic) Calcium Carbonate

### 2.1. The Interest in Obtaining Calcium Carbonate from Biological Species

Biological calcium carbonate has already received a considerable amount of attention, striving, in particular, to improve the sustainability of the materials’ production with inexpensive secondary raw materials, hence not affecting resource depletion [15]. Potential was also demonstrated in several applications, including, e.g., energy storage in batteries, considering its electrochemical stability [16]; water treatment and antibacterial action with silver nanoparticles [17]; the synthesis of hydroxyapatite for non-biomedical purposes, such as dye absorption [18]; and the production of bio-calcium oxide into industrial furnaces [19], or carbon-derived catalysts [20].

The role played by bio-calcium carbonate in composites and biomaterials depends on a number of factors, among which paramount importance is attributed to the structure (granulometry, geometry) of powders introduced into the matrices, which has an effect on their mechanical and thermal properties. Another factor, which strictly depends on the source, is the presence of the different allotropic forms of calcium carbonate (calcite and aragonite in the first instance, and also vaterite, when relevant, while portlandite is only generated in some cases during calcination) and their respective amounts. All the above factors contribute to the successful introduction of a sufficient amount of bio-derived calcium carbonate in the composites, and ultimately to the significance of such an operation. This is also noteworthy considering that the inclusion of calcium carbonate into polymer composites can also take some forms, such as nanospheres addition [21] or thin film layering [22], which are hardly reproducible using biogenic calcium carbonate, unless thermal treatment is applied after mechanical processing.

### 2.2. The Problem of Interspecies Differences in Biological Calcium Carbonate

To start clarifying the potential of introducing biological calcium carbonate as a substitution for limestone from quarries or other general mineral filler in composites, or as the replacement for natural hydroxyapatite in biomaterials, a substantial caveat needs to be raised concerning interspecies differences. In practice, when the aim of the study is the availability of a bare filler for composites, limited considerations (if any) of the very type of natural structure employed for it are normally made, for example, whereby studies generically refer to the introduction of “seashell waste” in polypropylene composites, such as in [23]. This is typical of sectors such as aquaculture, where the emphasis is rather placed on shells as a hazardous waste rather than a biological structure, so that applications in terms of energy recovery and fertilizers (biochar) can also be considered viable [24]. However, this does not impede its effective application in generic contexts when a thorough control of the granulometry is exerted; for example, clam powders with sizes between 125 and 149 μm, mixed with resin and cellulose, were able to improve the adhesion of plaster walls [25].

In Table 1, a number of biological sources of calcium carbonate from various species, which have been considered for their application in structural materials, are reported with some further characteristics from the literature. Those that will be treated more in depth, discussing their application in composites and biomaterials (Section 3), are emphasized by the use of bold italics.

In addition, it needs to be considered that, in some cases, specifically for mussels, the amount of calcite and aragonite depends on the region of the shell, varying, e.g., between the external prismatic layer and the internal nacreous one, which are arranged in cast tablets [36]. Moreover, for some species, such as the Mediterranean fan mussel (*Pinna nobilis*), the two forms of calcium carbonate appear to be physically distinguishable and separable. More specifically, calcite prisms are present on the outer layer, while the internal layer is made of aragonite nacreous tablets [37].

Polymorph separation is recognized as prospectively important, yet this exceeds the purpose of this review, which purposely concentrates on obtaining calcium carbonate with the objective of examining its introduction in composites and biomaterials. 

## 3. Application of Bio-Sources of Calcium Carbonate and Phosphate into Composite Materials and Biomaterials: Some Case Studies

### 3.1. Land Snail Shells

#### 3.1.1. General Considerations

The question of the use of land snail shells and relevant powders as a source of calcium carbonate and phosphate is critical insofar as the variability of the characteristics of the hierarchical structures in Gastropoda appear to be significant; this is also due to the very large number of species involved and their constructive variety [38]. From a biological taxonomy point of view, land snails shells, upon which this review concentrates, also bear some obvious relationships with those of seasnails. For completeness, it is worth mentioning that a number of studies on the potential applications of seasnail shells have also been performed. An attempt at the development of hydroxyapatite nanoceramics was carried out with chemical transformation using the hotplate stirring method [39]. Powder processing was also proposed, allowing copper (I) oxide (Cu_2_O) formation up to 3 wt.% in a lime–alumina (CaO·Al_2_O_3_) ceramic compound, where lime was obtained from calcium carbonate powder extracted from seasnail shells [40]. 

**Figure 3:**
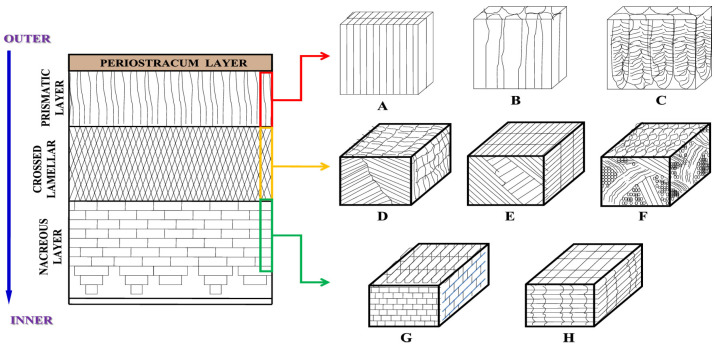
Arrangement of the three layers (prismatic, crossed-lamellar and nacreous) of the shells below the periostracum according to the species of seasnails. In particular, (**A**) lath-type fibrous prismatic layer (*B. bengalensis*), (**B**) blocky simple prismatic layer (*P. globosa*), (**C**) composite fibrillar prismatic layer (*B. costula*), (**D**) simple crossed-lamellar structure (*B. bengalensis*), (**E**) crossed-lamellar structure with larger lamellae perpendicular to the outer lamellae (*P. globosa*), (**F**) complex crossed-lamellar structure (*B. costula*), (**G**) nacreous layer with typical brick-and-mortar wall model (*B. costula*) and (**H**) row stacking nacreous layer (*B. bengalensis* and *P. globosa*). Insets indicate the orientation of calcium carbonate crystals in the shells of the respective snails: prismatic layer is indicated by the red arrow, crossed lamellar by the yellow arrow, and nacreous layer by the green one.

In particular, this has been conducted using simple chemical treatments with orthophosphoric acid (H_3_PO_4_), such as on tropical seasnails (*Cypraea tigris*), which have a characteristic structure including clipped fibers [41]. However, the disposition of calcium carbonate crystals may take different forms in snail shells. In [42], a number of fibrous arrangements were identified, depending on three different species of freshwater snails, namely *Ellamya bengalensis*, *Pila globosa* and *Brotia costula*. The study of these three different seasnail species includes prismatic layers, simple and complex crossed-lamellar structures, and nacreous brick-and-mortar layers, as depicted in Figure 3.

As far as land snails are concerned, some aspects are especially crucial in revealing the dependence of the properties on the very hierarchical structure of the shell, such as in the case of its water retention capabilities. This was demonstrated to be strongly influenced by surface roughness; only a 10-micron maximum peak-valley roughness was really able to modify the dynamic contact angle of water droplets [43]. Such simulation studies were intended to provide simple models of the interspecies differences. The ability of snail shells to repel both oil and water, therefore to be superamphiphobic, has attracted some interest for the fabrication of coatings inspired by these structures. This attention has been especially based on the coexistence of hard shells and soft epiphragms in snail exoskeletons [44]. This is unlikely to be of interest when power-form snail shells of less than a 50-micron granulometry are used, yet it might be suggestive of future developments involving the controlled introduction of snail shell particles as filler.

#### 3.1.2. Applications of Snail Shells in Composites

The application of snail shells in composites does sometimes appear to be focused on the peculiar properties obtained from one species, as has been the case for Archachatina marginata shells in aluminum alloys and obtaining metal matrix composites. In this case, snail shell powder has been employed in its calcinated form, under temperatures up to 900 °C, in order to exploit the hardness obtainable via the presence of various oxides in the material [45].

In most cases, however, the species factor was considered rather negligible in applications; researchers choose, rather, to concentrate on the geometrical characteristics or else, in the case of snail shell powders, the granulometric characteristics. The non-specific application of snail shell powder was proposed by Karaoui et al., who tested wt/wt% variations of snail shell powder (SSP) up to 63 µm in size in polystyrene (PS) composites up to 10 wt.% SSP in order to observe the resulting thermal, mechanical, and structural differences [46]. The powder was obtained from shells purified using distilled water and chlorine bleach, then ground in a ball mill and calcinated to obtain stable CaO/CaCO_3_ phases. The addition of the maximum amount of SSP offered the highest crystallization temperature of polystyrene, bringing it to around 180 °C with a crystalline content of around 20%. This proves that SSP acts as a nucleating agent, hence increasing the number of sites for the initiation of the process; on the other hand, the viscosity of the composite is increased by the addition of SSP, which further complicates material processing, a critical factor when using thermoplastic matrices. Another study by Okafor et al. concentrated on the possible introduction of SSP into low-density polyethylene (LDPE); in this case, a powder medium granulometry of 83 µm was used with, again, a maximum content of 10 wt.% by pelletizing the powder with the polymer [47]. The introduction of the maximum amount of SSP offered an increase in tensile strength of around 10% with respect to neat LDPE (from 128.29 to 137.56 MPa), though with some inconsistencies for lower amounts of SSP. This increase was further significant for flexural strength (from 120.34 to 145.76 MPa), where it exceeded 20% for 10 wt.% SSP, and with more graduality.

Also, in thermosetting composites, the use of SSP was proven to be useful. Some operations do appear debatable, such as the introduction of amounts of up to 15 wt.% of snail shell powder (SSP) into bisphenol A (BPA)-based epoxy resin composites. Apart from the increase in glass transition temperature and degradation temperature, the properties were degraded after the introduction of more than 10 wt.% of SSP, which was attributed to poor compatibility between the resin and the powder. The heat treatment of SSP at 800 °C did not result in any particular advantage [48]. Conversely, Gbadeyan et al. aimed to exploit the calcium carbonate content of snail shells to synthesize nano-fillers for epoxy composites [49]. Following a critical evaluation of the filler particle sizes required to enhance the composite strength, the group decided to develop nano-CaCO_3_ as a filler for epoxy resin matrix, using a dual dry and wet milling procedure to produce a particle size of ≤50 µm. The resin casting method was used to prepare the different composites containing 1–7 wt.% nano-CaCO_3_. Concerning mechanical properties, the composite with 1 wt.% SSP showed the highest tensile strength of 78 MPa and a relevant stiffness of 6 GPa. The strength progressively decreased with increasing SSP concentration, possibly due to the non-uniform filler distribution, while, in contrast, the stiffness was rather fluctuating, though, again, the best value was obtained for the lowest filler content. It was concluded that the homogeneity provided by the 1 wt.% concentration was unmatched in higher concentrations.

Ghadeyan et al. concentrated on obtaining calcium carbonate from a particular type of snail, the giant African land snail (*Achatina fulica*), by optimizing micromilling to below 50 microns [50]. Researchers from the same group introduced up to 20 wt.% Achatina fulica SSP into an epoxy matrix, finding improvement in various properties, even in the tensile strength, and investigated the production of hybrid composites with eggshell powder, proving the existence of a synergistic effect with the combined introduction of the two fillers [51].

#### 3.1.3. Applications of Snail Shells in Biomaterials

**Figure 4:**
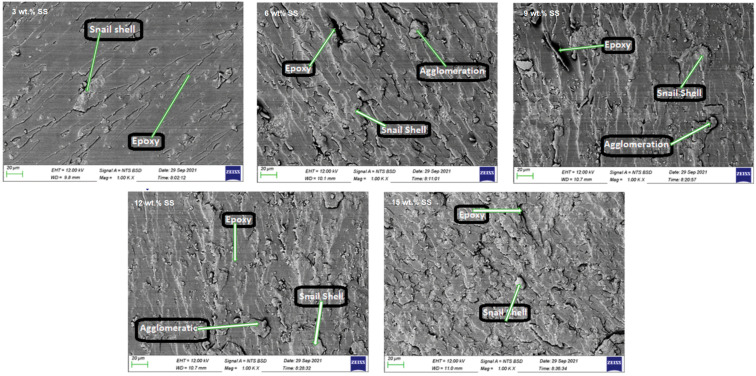
Epoxy bio-composite samples with varying wt.% snail shell (SS)-derived hydroxyapatite (HAp) [52].

Also, in the case of snail shell powder, its use in its calcinated form as a source of hydroxyapatite (HA) was investigated. In particular, Oladele et al. fabricated epoxy-based composites with up to 15 wt.% snail shell-derived HA by stir cast molding, with the idea of them serving as adhesive biomaterials; they measured the flexural strength, hardness, impact, thermal conductivity, and wear index [52]. The different levels of adhesion and interface quality with increasing HA are reported in Figure 4. The improvement in the properties was rather consistent with the increase in HA, although the geometry and the size of these was very variable, spanning from a few microns to over 0.1 mm.

Another biomaterial application with snail shell powder concerned the production of HA/l-lysine composites. These composites were employed to modify a carbon glassy electrode and effectively improved electronic transfer, as verified by using it in the electroanalysis of toluidine blue in water [53]. More evolved applications in geometrical terms have been also attempted to synthetize HA nanorods from snail shells, which would ease implantation; this was obtained by microwave irradiation through a suitable chelating agent, based on ethylenediaminetetraacetic acid (EDTA) [54]. More information on the biological activity of snail shell-obtained HA was also elucidated, including anti-microbial, anti-biofilm and biocompatibility tests on specific snail (*Atactodea glabrata*) shells [55]. Further studies on biocompatibility are available for garden snail shells (*Helix aspersa*) with high bioactivity due to the surface formation of apatite [56].

A general overview of the production of calcium carbonate and hydroxyapatite from snail shells suggests that the species employed are the most different among them, with selection being based, so far, on local availability. Despite this, it is also fair to say that limited comparative studies among snail species exist that would shed some light on their respective merits in the process.

### 3.2. Clam Shells

#### 3.2.1. General Considerations

Investigations into the mechanical properties of clam shells have been particularly significant over the last decades, involving a number of comparative studies, such as [57], which involves the evaluation of the giant clam’s (*Tridacna gigas*) shell mechanical properties against conch (*Strombus gigas*) and red abalone (*Haliotis rufescens*). As is possibly expected, giant clam do not compare favorably with the other two mollusks in these terms. In smaller clam shells, such as that of the Manila clam (*Ruditapes Philippinarum*), the three-layer pure aragonite structure is more obvious, which is disposed in three partially overlapped brick-and-mortar layers. As a consequence of this nacreous structure, the preferential orientation of hardness in the middle and outer layer has also been elicited [58]. A characteristic that is particularly beneficial for the mechanical performance of this nano-hierarchized structure is the creation of an inner electrical field as a consequence of material self-assembly. This results in piezoelectricity and, therefore, the capability of being deformed under the application of a generated electrical current [59]. In another sense, the large variability in size and also the static friction coefficient of clam shells on different materials have also been investigated to improve the mechanical processing of clam shells into powders of various granulometries [60]. The flexibility of use and the large availability of clam shells also allowed them to be used as a precursor, together with soda lime glass waste, for the formation of glass ionomer cement, i.e., a mixture of glass and an organic acid, for prospective use in dentistry [61]. Clam shells also possibly represent the most viable source of biogenic calcium carbonate for reasonably easy application in the fields of both polymer and cement composites. This is namely for their mechanical effect on cement performance, confirmed for compression strengths up to 8 wt.%, and less so for their splitting tensile strength when working with calcinated clam shells; this property decreased from 5.1 to 4.4 MPa with the introduction of 6 wt.% clams [62]. Also, in the case of clams, mixtures of shells from different species, e.g., cockles and Manila clams, are often used in cement, which are only controlled ex-post based on required properties, such as water retention, strength and interfacial adhesion with mortar [63].

#### 3.2.2. Applications of Clam Shells in Composites

The introduction of clam shell powder (CSP) into sustainable composites proved more difficult due to variety of species and to their substantial contamination in the food-related sector. In some cases, the attention paid to their technical value, starting from locally available clams, proved their introduction to be successful. This was effective, for example, for the compression strength and Vickers hardness of unsaturated polyester in the case of *Polymesoda bengalensis*, which is graded into eight different average granulometries [64]. The results showed that the hardness of the unsaturated polyester improved as the CaCO_3_ filler was infused into the matrix. An attempt to facilitate this process was carried out via the treatment of clam shell powder using furfural; in this way, CSP was proposed as a replacement filler for calcium carbonate in polypropylene matrix composites, but in higher amounts (15 vs. 10 wt.%) to obtain comparable results [65]. In terms of the potential of clam structures, more results appear to be possibly obtainable, in light of their large flexibility for various uses; this has been proven applicable in different fields. A caveat in their application as filler is the interspecies differences in calcium carbonate among clam shells. As an example, granular structures were found in brown clamshells (*Meretrix meretrix*), whereas agglomerates were revealed in grey clamshells (*Meretrix casta*), which would produce considerable differences from a tribological point of view and would hence be reflected in the filler–matrix adhesion [66].

A good example is represented by the possibility of cold sintering for ball mill-pulverized clam and oyster shells, sieved to 63 μm with uniaxial pressure and temperatures below 300 °C, to allow densification and lead to their possible application as construction materials. Observations by X-ray diffraction (XRD) indicated the modalities of phase transformation from aragonite to calcite under temperatures lower than 300 °C. Cold sintering decreases the amount of residual water, whilst, on the other hand, compression strength declines at 200 °C, since water promotes densification [67].

More substantial heating treatment up to calcination, which normally requires a temperature of 1350 °C for pure cement clinker, has also been applied, however, for the successful incorporation of clam shells into construction materials; in this case, the crushing of clam shells is performed at dimensions smaller than a 200 mesh (approximately 74 microns). The optimal baking temperature was calculated according to the residual calcium oxide content, which must be lower than 1.5 wt.%; the results indicated that the optimum temperature is 1400 °C with clam shells, reducing the baking temperature for synthesis. For the five cementitious clinkers examined, in all cases, clam shells improved the compression and flexural performance, as the result of increasing the amount of tricalcium silicate while reducing that of dicalcium silicate [68].

#### 3.2.3. Applications of Clam Shells in Biomaterials

The possibility of synthesizing hydroxyapatite from clam shells has also generated considerable interest, as reviewed in [69]. Various experimental routes were employed for this purpose, including hydrothermal, sol-gel, mechanochemical, precipitation and wet chemical methods. In particular, the hydrothermal method, at temperatures in the region of 140 °C via autoclaving for 12 h, was proposed in [70] to obtain hydroxyapatite nanopowders with a uniform wire-like geometry. Hydroxyapatite obtained from clam shells has been proposed for various purposes, and not exclusively biomedical ones. This includes water bioremediation via the removal of heavy metals, more specifically bivalent Pb(II), Cd(II) and Cu(II) ions [69], yet also some potential applications for biomedical purposes, such as for bone implants, which require hydrothermal treatment at higher temperatures (200 °C) [71]. In the latter case, the strontium doping of Mercenaria clam shells for hydroxyapatite synthesis has also been proposed, which is known to promote osteoblast replication [72].

**Figure 5:**
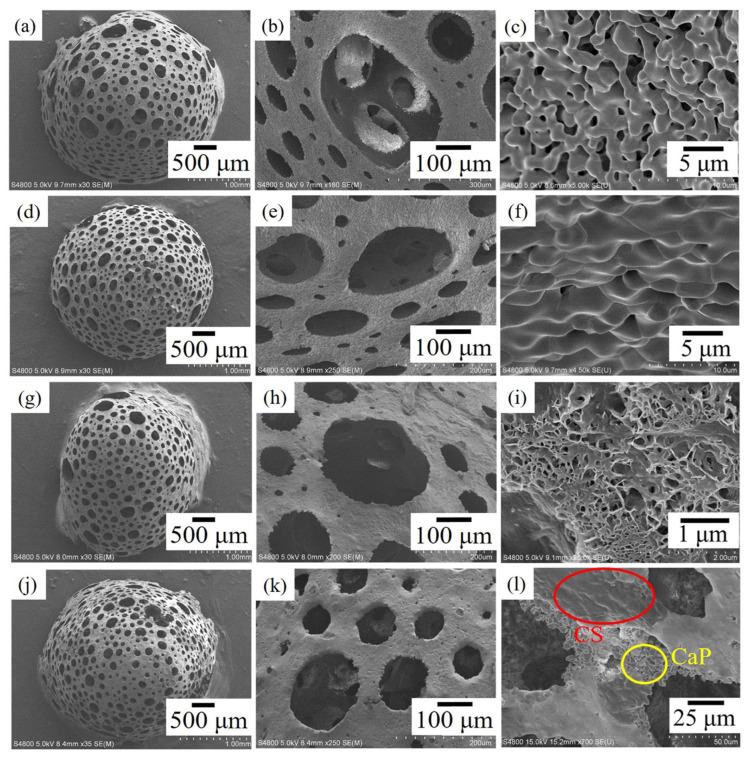
Field-emission scanning electron miscroscopy (FE-SEM) images of porous calcium phosphate (Ca_3_(PO_4_)_2_) granules. (**a**–**c**) Furnace-cooled (FC), (**d**–**f**) air-cooled (AC), (**g**–**i**) FC/Chitosan-coated (CS) and (**j**–**l**) AC/CS with identified calcium phosphate porous granules (CaP) [74].

In particular, the gas-foaming technique enabled the synthesis of hydroxyapatite (HA) powder and porous calcium phosphate (TCP) granules from clam shells ground down to 200 mesh. Their combined use would couple the higher biocompatibility of HA with the easier biodegradability of TCP. The trace elements present in clam shells, namely zinc, magnesium and strontium, also promote the growth of bone tissue [73]. The combination of the aforementioned porous granules obtained from clam shells through gas-foaming using inert gas with other biological materials, namely chitosan from crab exoskeletons, improved the mechanical properties of the foam by concealing its surface defects. Furnace-cooling (FC) produced biphasic hydroxyapatite/β-tricalcium phosphate granules, while air-cooling (AC) offered triphasic hydroxyapatite/β-tricalcium phosphate/α-tricalcium phosphate granules. The latter showed a 79% higher compression strength, passing from 520 to 930 kPa, which was then improved by 21% up to 1125 kPa through chitosan coating without affecting its biocompatibility. Under all cooling conditions, the porous granules exhibited macropores (>100 µm) and micropores (<100 µm), and the porosity exceeded 82% [74]. The various porosity dispositions are reported in Figure 5. Also, regarding the biomedical applications of nano-hydroxyapatite from clams (Meretrix meretrix), in vitro studies were also carried out, focused on the negative control and carried out using the erythromycin antibiotic, against *Staphylococcus aureus* and Pseudomonas bacterial strains [75].

As far as the other methods are concerned, work on the mechanochemical synthesis of hydroxyapatite from mercenaria clam shells led to a product with crystallinity in excess of 85% and a crystallite size in the 50–70 nm range, which is much higher than in most other cases [76]. In contrast, using the conventional wet chemical precipitation method, normally carried out in an alkaline environment at a pH of around 10, followed by a sintering stage to bring the crystallite size to the previously indicated range, a 1100 °C processing temperature was required. This was obtained in a study based on waste from ark clam (*Anadara granosa*) shells, although it is also possible for a number of other biological sources of calcium carbonate [77]. Ark clams were promising for the purpose in the sense of yielding Ca/P ratios closer to the stoichiometric ratio (1.67), closer than is the case for Asiatic hard clams (*Meretrix meretrix*) (1.92 vs. 2.13) [78].

Very recently, to better exploit the bioactivity of clam-extracted hydroxyapatite, its application as a 300-micron coating layer over a biocompatible titanium alloy (Ti-6 Al-4 V) was also proposed [79]. Expanding the context of effective body tolerance, HAP from clam shells was also proposed as a substitute for avobenzone and oxybenzone in sunscreen products, resulting in the effective absorption of UV rays, combined with an absence of adverse skin reaction, a result that is promising for further applications in cosmetics [80].

### 3.3. Mussel Shells

#### 3.3.1. General Considerations

**Figure 6:**
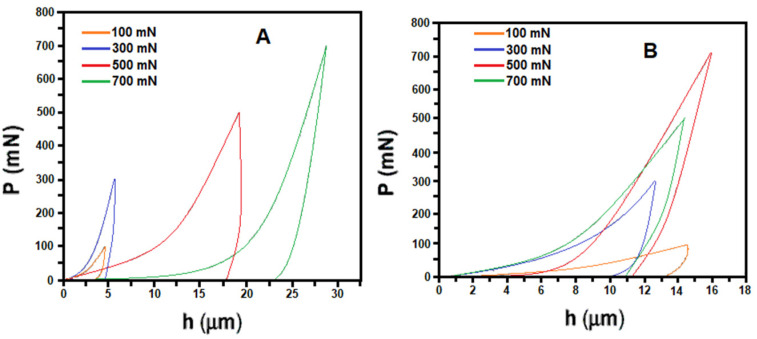
Typical load depth (P–h) plots from nanoindentation experiments conducted on mussel shells: (**A**) *Corbicula bensoni* and (**B**) *Lamellidens marginalis* [82].

Most waste mussel shells that have been considered in studies for obtaining bio-calcium carbonate belong to the same species, *Mitilus galloprovincialis*, which has become a sort of standard in the European tradition, both for food applications and for its recognized filtration ability [81]. It is worth noting that different species are encountered in materials research: wider research interest is particularly garnered due to the variable characteristics of the obtainable calcium carbonate. Interspecies studies elucidating the different characteristics of calcium carbonate, and also their potential for use in a powdered or calcinated form, have also been conducted, e.g., in [82], in which the authors considered *Corbicula bensoni* and *Lamellidens marginalis* as representative species. The CaCO_3_ content of the shells (up to 95–96% of the shell weight) of both the mussels was positively correlated with the shell length, suggesting an increased deposition of CaCO_3_ in shells with the growth of the species. The cross-sectioned views of the shells exhibited a distinct layered structure with an external periostracum and an inner nacreous layer varying distinctly with prominent growth lines. The combined presence of calcite and aragonite offered, in this case, significant strength at the nanoparticle level of the shells, with a clear effect of stiffness increase and, hence, densification; the relevant curves are reported in Figure 6.

For example, regarding the shells of green mussels (*Perna viridis*), which are cultivated and endemic in the Indian region [83], a comparison between the characteristics of its calcium carbonate structure and the commercial one proved that both are quite identical. Moreover, its predominance of vaterite crystal phases (up to 91.2 wt.%) and its virtual absence of impurities provided a distinct advantage for its application in biomaterials in terms of non-toxicity and biocompatibility [84]. Vaterite, which is less stable than calcite and aragonite, is seldom found in mollusk shells, and its presence is due to unusual phenomena such as remineralization or the initial stages of shell formation [85]. A typical environment in which vaterite is synthesized is farmed fish otoliths, which are subjected to fast growth and therefore to the formation of this calcium carbonate form, which is normally only transient in the organism’s life [86].

#### 3.3.2. Applications of Mussel Shells in Composites

Also, the introduction of mussel shell powder in polymer composites and biomaterials has been attempted by a large number of researchers, again starting from investigations into the biomineralization process [87]. As has typically been shown in the recent literature on waste-filled composites, epoxy resins have been often employed for their recognized reliability. In this sense, mussel shells have also been compared with two types of ligneous fillers, namely hazelnut and walnut shell chars, regarding water sorption, fire resistance and thermal performance and introducing up to 50 wt.% mussel shell filler [88]. The difficulties of this process were revealed through the considerable variability of granulometry realized in milling processes; in particular, in [89] the average diameter of particles was 74 μm with a considerable standard deviation, to a level of 60 μm, which effectively indicates this sort of issue. It is suggested that varying the type of epoxy, including bio-formulations, used to produce the mussel shell-reinforced composites would not result in a particular effect over composite performance, indicating its consistent growth up to 50 wt.% filler [90].

**Figure 7:**
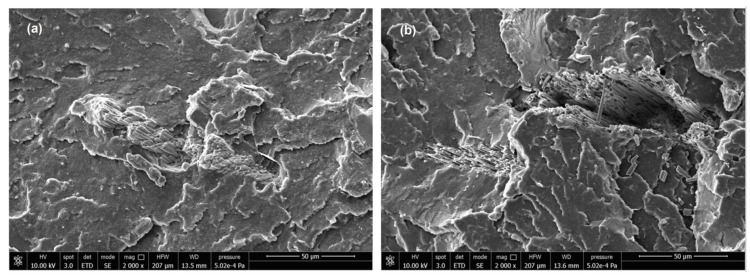
Presence of interface cracks in biocomposites with mussel shells (2000× magnification): (**a**) 10 wt.% shells; (**b**) 20 wt.% shells [91].

The more refined fragmentation, down to a few microns, of mussel shells also suggested the possibility of shifting to bio-based matrices, such as poly(lactic acid) (PLA) and polybutylene adipate-co-terephthalate (PBAT), obtaining a performance comparable to that of calcium carbonate extracted from quarries [91], although some interface issues have been also highlighted, as shown in Figure 7. To move onto the use of thermoplastic matrices via the thermal improvement obtained by the application of powder mussel shells, their introduction in the measure of 5 wt.% was proposed for polypropylene filaments produced by melt mixing or extrusion [92]. However, other attempts have also been carried out to introduce mussel shell powder (up to 10 wt.%) with different thermoplastic polymers, as is the case for polyphenylene sulfide (PPS), where tribological properties were particularly improved at 30° [93].

Mussel shells are also among the few biogenic calcium carbonate structures whose use has been proposed in real products, e.g., for the transportation industry, for example, in the case for motorcycle clutch linings [94].

#### 3.3.3. Applications of Mussel Shells in Biomaterials

Calcium carbonate from waste mussel shells has also been proposed as a secondary raw material for pharmaceutical applications, in particular for its treatment with levulinic acid to obtain calcium levulinate. This also allowed for the assessment of its low toxicity via the Constantinescu phytobiological method, employing wheat kernels (Triticum vulgare Mill) [95]. Another possibility that has been investigated is the proposition of mussel shell calcium carbonate for the synthesis of hydroxyapatite (HA) to obtain a bone replacement material. HA nanopowders have been consolidated into cylindrical pellets by uniaxial pressing and sintering between 800 and 1100 °C. The phase composition of the sintered materials depends on the Ca/P molar ratio and on the specific CaCO_3_ source, most probably due to the presence of different ionic species and also to the variable densification and sintering temperatures. Preliminary in vitro tests did not reveal any cytotoxic effects, whereas good cell adhesion and proliferation was noticed at day 1, 3 and 5 after seeding. Among the different calcium carbonate sources, mussel-derived HA also well supported cell adhesion [96]. In a more functionally elevated sense, calcined mussel shell was also used with the idea of developing a visible light (λ > 420 nm)-driven photocatalyst nanocomposite including only 5% bismuth molibdate (Bi_2_MoO_6_), where the maximum catalytic effectiveness was obtained. In view of the reduced cost of the secondary raw material, this could be used for water treatment [97]. A hydrothermal method enabled a reduction in calcination temperature to 900 °C when extracting hydroxyapatite from green mussel shells, though this lead to the formation of a considerable amount of calcium hydroxide mineral, also referred to as portlandite (24.7%) [98].

Also in the case of mussel shells, the rapid microwave method was applied to obtain highly nanocrystalline hydroxyapatite using EDTA as the chelating agent [99]. This proved to be particularly well adapted for potential biomedical applications, yielding nanoparticles of 30–70 nm very quickly (in no longer than 30 min). This is particularly significant when compared to the more traditional yet lengthy method of wet slurry precipitation. The latter was also experimented on through the use of monopotassium phosphate (KH_2_PO_4_), with the aim of producing a catalyst for azo-dye removal in water treatment, which allowed for a 62% degradation of methylene blue in 24 h [100]. Most recently, an alternative use of hydroxyapatite from green mussel (*Perna viridis*) shells was also proposed in using it to coat AISI316 steel for the surface modification of biomedical stainless steel using a spray gun preheated to temperatures of up to 600 °C [101]. This offered accurate thickness control with an accuracy lower than a micron, and will also be prospectively developed in the future for different biomedical alloys, such as Ti-6Al-4V, Co-Cr or Co-Cr-Mo.

### 3.4. Oyster Shells

#### 3.4.1. Application of Oyster Shells in Composites

**Figure 8:**
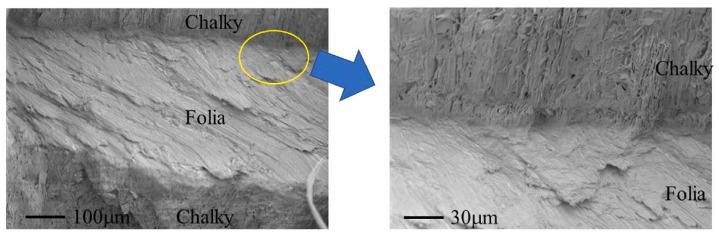
Microstructures of the folia and chalky layers: the right image represents an enlargement, as indicated by the circle, of the left one [103]. The arrow indicates from which region the inset was taken.

Regarding oyster shells, due to their specific properties (very high levels of calcite and capability of obtaining complex carbonatic structures, especially from the shells of juvenile exemplars [102]), though the amounts of waste available are lower than in the case of clams and mussels due to the smaller market, the possibilities are multifold. The microstructures of oyster layers comprise lamellar (folia) and sharper (chalky) characteristics, as indicated in Figure 8 [103]; the former are around 400 μm thick, while the latter range from 250 to 300 μm.

Apart from the initial attempts at introducing oyster shell powder in construction materials, which have also recently been implemented at a European level [104], only some studies have concentrated more on the use of oyster shell powder (OSP) for the reinforcement of polymers. A typical case was applying OSP in polypropylene through an injection molding process and comparing it with mussel shells. An amount of 10 wt.% CaCO_3_ from either source did not bring any variation in melting temperature for the polymer and the tensile and impact performance also compared well with samples with the same amount of CaCO_3_. The only limitation was in terms of elongation to break, which was considerably reduced with bio-calcium carbonate, which was attributed to the not entirely ineffective control of the granulometry [105]. However, the aforementioned issues suggest the structural complexity of oysters can be better exploited; local investigations have been carried out, such as in the case of Brazilian production in mariculture, centered on the district of Florianopolis [106]. Moreover, oyster shells also show some potential in providing further properties for the penetration of thermoplastics, such as polypropylene, in other fields, for example, to confer antibacterial properties [107].

However, the possibility of fragmenting oyster shells into powders of very small dimensions, even in the region of 200 nm, allowed their introduction in large amounts (up to 30 wt.%) in rubber matrix composites. In this way, researchers were able to produce a significant improvement of the tear strength (maximum advantage 27.9% for 25 wt.% of oyster shell modified powder) [108]. The thermal treatment of oyster shell powder at 600 °C also allowed the obtaining of a high specific area (SA), enhancing its porosity; the SA increased from 5.8 to 12.7 g/m^2^ [109]. This allowed for its introduction into a polycaprolactone matrix (PCL), which was also successfully tested against *Staphilococcus aureus* and *Escherichia coli*, proving that the larger amount of thermally treated powder introduced also increased the antibacterial effect of the polymer.

#### 3.4.2. Application of Oyster Shells in Biomaterials

Another possibility is the use of oyster shells as a possible source of hydroxyapatite for bones, considering the potential of the latter in terms of bone reconstruction applications [110]. This is particularly interesting in light of the limited contamination of oyster shells, which suggests the effectiveness of sintering processes at very high temperatures (1000 °C) [111]. This can also be realized in light of its potential applications for bone tissue engineering, for example, by using shells from *Crassostrea angulata* oyster. Hydrothermal conversion is recognized as providing hydroxyapatite from calcium carbonate crystals without destroying the raw material morphology through high temperatures or pressure [112]. By applying a temperature of 220 °C for 6 h to a mixture with a Ca/P molar ratio of 5/6 and with 50 mL of deionized water in an autoclave environment, plate-like nanocrystals of AB-type carbonated hydroxyapatite (HA) were formed. The polymer replication method, based on templating the material around and in the pores of a polyurethane sponge [113], allowed these macroporous HA nano-powders to develop a bone tissue scaffold (O-HA). Furthermore, a comparative study was performed using commercially obtained HA nano-powders (S-HA). It was highlighted that oyster shell scaffolds exhibited better cellular biocompatibility than their commercially obtained counterpart, together with a high plasticity and more easily controllable pore size [114]. Another possibility that has been explored concerned the formation of blends including hydroxyapatite powder and calcium carbonate from pearl oysters (*Pinctada maxima*). The primary idea is to exploit the nacre structure characteristics with the aim of overcoming the load-bearing limits of hydroxyapatite [115]. To improve the compatibility between the two ceramic structures, various surface treatments were applied on nacre, of which the most effective proved to be sodium hypochlorite, which lead to the removal of loose or less structural material. However, treatments offering coating protection on nacre have also been attempted, such as in the case of aminopropyltriethoxysilane (APS) compatibilized on the surface through the action of solvents, particularly ethanol or toluene [116]. Further work aimed at effective scaffolding from oyster shells powder was also carried out using a hybrid biomaterial comprising alginate and hydroxypropyl trimethyl ammonium chloride chitosan to provide mutual electrostatic action [117]. The expansion of the biocompatible possibilities was also recently proposed through the addition of a 2% of carbonate hydroxyapatite from oyster shells to poly(methylmetacrylate) (PMMA). The material, with a crystallite size in the region of 70 nm, was then immersed for 30 days in simulated body fluid [118].

### 3.5. Eggshells

#### 3.5.1. General Considerations

The case of eggshells is significant, on the one hand, since the variety of biotechnological possibilities they offer is much wider and more differentiated than is the case for other sources; they might also serve as general inspiration [119]. There is also active research into keeping the thickness of chicken eggshells as constant as possible, normally in the region of 300 microns, which represents a factor related to the quality of the product; this is, therefore, considerably sought after [120]. This also offers relevant advantages for biogenic calcium carbonate in terms of higher reliability and the limited scattering of the supply properties.

The possible applications of eggshell powder (ESP) in materials, other than for polymer composites and biomaterials, and even excluding those in the food sector, are multifold; as such, they can only be skimmed over in this review. These include the following:

1. Production of active carbons

This application was assisted by the typical hexagonal geometry of eggshells, which was then activated with the help of orthophosphoric acid (H_3_PO_4_) [121]. As is the case for other biogenic calcium carbonate sources, the obtained active carbons are particularly effective in the case of dye removal [122], although the introduction of eggshell powder in other active carbons would also offer some results in terms reducing the heavy metal content during water treatment [123].

2. Production of catalysts for biodiesel

Eggshells have been used as moderately alkaline catalysts for the production of biodiesel from soy oil with calcination at 900 °C for 3 h [124]. This was attempted even with secondary raw materials, such as waste cooking oil, where eggshell powder provided multimode calcium oxide by calcination at 800 °C for 2 h [125].

3. Various engineering materials

**Figure 9:**
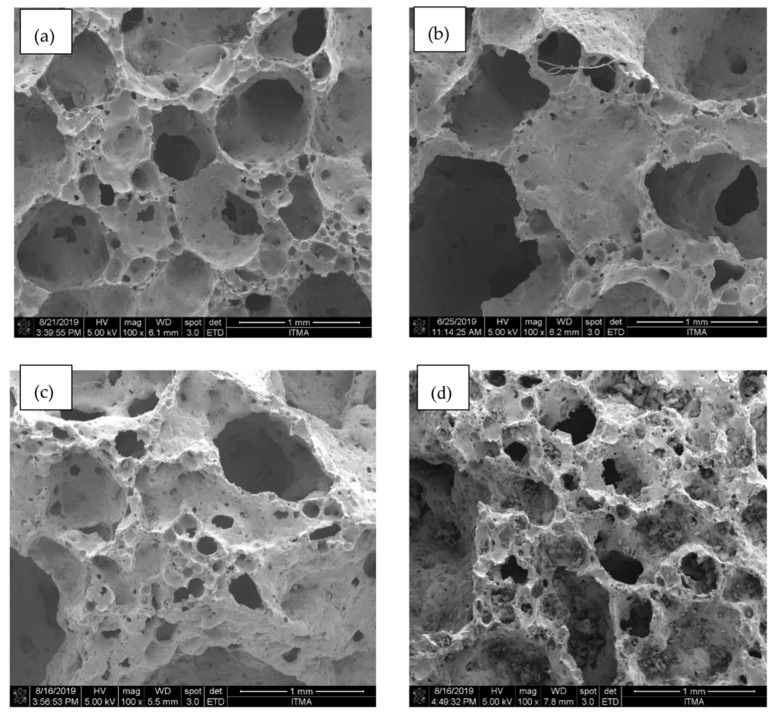
Microstructure of foam glass-ceramics with (**a**) 1 wt.% eggshell powder (ESP); (**b**) 3 wt.% ESP; (**c**) 6 wt.%; and (**d**) 9 wt.% ESP [126].

The repeatable and light structure of eggshells has also been exploited in the production of porous glass-ceramics, to amounts of up to 9 wt.%, adequately tailoring porosities (Figure 9) [126]. Other uses more suited to combinations of lignocellulosic materials are in the production of sustainable drywall (in a hybrid with sugarcane bagasse waste) [127] and as filler in wood–plastic composites [128].

#### 3.5.2. Applications in Composites

Eggshell powder (ESP), in light of its abundance in food waste, its very large amount of calcium carbonate, the facile separation of its protein content, and its controllable fineness post grinding, has been proposed in the last two decades as an ideal filler for industrial thermoplastics such as polypropylene [129]. Its wide use as a hardener and a rheology controller, as an alternative, e.g., to industrial calcium carbonate and talc, in thermoplastic polymers, such as polyolefins, is well known and explored [130]. Regarding its mechanical properties, these appear to be usually enhanced by the introduction of ESP, although not all studies are in agreement on this outcome. Recent expansions of the use of ESPs also concern their application as fillers in aluminum alloys to improve friction resistance properties [131]. Some moderately innovative expansions of this procedure and concept have also found applications, such as in the possible manufacturing of biodegradable films based on polypropylene carbonate (PPC), where filling with up to 4 wt.% ESP resulted in a positive effect over the tensile performance of the film [132]. It is fair to say that the use of ESP can be reported to span over the whole field of composites, such as in metal matrix composites, as discussed in a previous review [133], resulting in its application as an adsorbent, catalyst, additive, and functional material. A number of factors appear critical in this respect: these include filler loading, particle size, and the filler/matrix interaction, with the possible addition of compatibilizers to enhance bonding and accelerate curing rates. Eggshells have also been considered as a possible source for hydroxyapatite production. The thermal stability of HA obtained from eggshells proved to be inferior, degrading below 800 °C, in comparison to that obtained from other food waste sources, namely bovine bone and fishbone, though the Ca/P ratio is, in all cases, considerably higher than the stoichiometric ratio typically encountered in human bone, i.e., 1.67 [134].

It is, therefore, worth mentioning that the possible applications of this very largely available waste, with its simple and repeatable structure, even expand to sectors that are not, strictly speaking, of interest in terms of the other sources of calcium products considered in this review. This is the case, for example, with the antibacterial use of ESP, which is fostered when thorough decontamination and deproteinization is applied to this product for its use in the food industry. This is considered in [135], where the authors used eggshell powder heated for 20 min at 60 °C (HESP), found that it exhibited antimicrobial activity against bacterial cells, fungi and bacterial spores, and determined the minimum inhibitory concentration (MIC) using kinetic analysis. HESP was found to effectively kill Bacillus subtilis spores to the level achieved with scallop shells powder. The study also observed the generation of active oxygen species from HESP, indicating its potential as a natural antimicrobial agent. The findings suggest that HESP could be utilized for antimicrobial purposes due to its high pH and active oxygen species.

#### 3.5.3. Applications of Eggshells as a Source of Hydroxyapatite

Due to the previously mentioned abundance of eggshells as waste, and due to the relative ease of processing the obtained powder, a number of methods were employed in light of the production of synthetic hydroxyapatite, which, conversely, generated powder with different morphologies. These include the microwave rapid method [136], precipitation through phosphoric acid [137], a solid state reaction [138], the hydrothermal method with ammonium phosphate [139], and agglomeration crystallization; in the last case, egg shells are used as the template for the growth of hydroxyapatite crystals [140]. It has also been found that calcination at higher temperatures up to 1200 °C lead to the very elevated interconnected porosity of eggshell powder, up to 73% [141]. The specific 3D arrangement of eggshell powder also represents an opportunity to provide a scaffold structure for the development of nanoparticles. This appears of interest for pulp regeneration in the dental repair field, in combination with collagen and epigallocatechin gallate (EGCG) [142]. In more general terms, eggshells are also being increasingly considered for hydroxyapatite from a bone grafting perspective, which justifies recent studies on their potential when Ti-doped, showing a considerable development of osteoblastic and osteoclastic cells, therefore balancing bone resorption and new formation [143].

### 3.6. Cuttlefish Bone

#### 3.6.1. General Considerations

The inner structure of cuttlefish bones consists of a network of interconnected chambers called septa, which provide buoyancy to the fish. Recent research has focused on utilizing these properties to create composites with improved mechanical strength, flexibility and lightweight characteristics. By incorporating the cuttlefish bone powder or nanomaterials derived from it into various matrices, such as polymers or ceramics, researchers have achieved remarkable enhancements in material performance.

**Figure 10:**
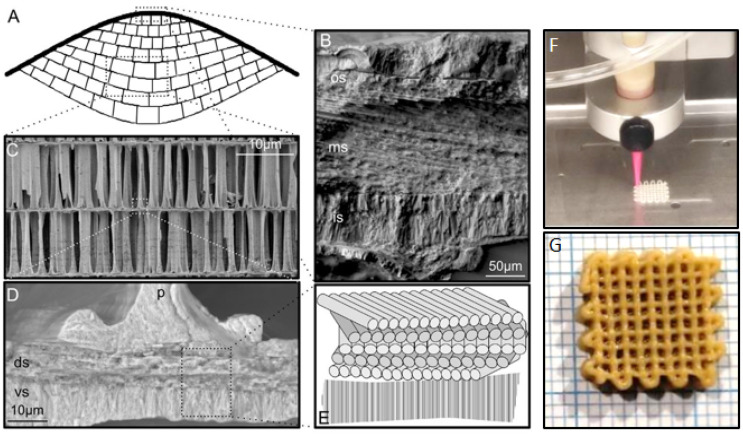
(**A**) Schematic cross-section of the cuttlebone, indicating the dorsal shield (thick black line) and the chambers (cellular network, thin black lines), which consist of septa and pillars. Scanning electron micrographs of (**B**) the three distinct layers in the dorsal shield, (**C**) the pillars supporting the adjacent chambers and (**D**) the intersection of a septum and pillar highlighting the different microstructures within a septum. In this sub-figure, p = pillar, ds = dorsal septum and vs = ventral septum. (**E**) Schematic of the change in direction of fibers in the septum [144]; (**F**) an example of 3D-printing with cuttlebone composite-based inks; and (**G**) the final composite after dehydration with phosphate-buffered saline [147].

Cuttlebone is not just a homogeneous and chemically isotropic composite; there are significant chemical and mechanical variations that were revealed utilizing attenuated total internal reflection (ATM), Fourier transform infrared (FTIR) and microCT (computer tomography) methods (Figure 10A–E) [144]. Cuttlebone has a highly organized internal shell structure constructed from aragonite in association with an α-chitin organic framework [145]. Also, SEM studies showed that cuttlebone shell has a chamber-like architecture (porosity, 93 vol.%; specific gravity, 0.19) in the form of mineralized sheets arranged in parallel layers and separated by S-shaped pillars, which functions as a rigid buoyancy tank that enables the animal to withstand external pressures of up to 2.4 MPa and maintain a fixed position up to a depth of 230 m [146]. This coexistence of strong rheological parameters and a light weight in cuttlefish bone not only provokes the use of this biologically derived material itself, but also the construction of new structures with predetermined parameters de novo. Three-dimensional constructing blocks inspired by cuttlefish bone lattices were optimized by computer simulations utilizing the ANSYS software package as a theoretical model for understanding and reproducing the base structure [147].

The combination of weak and hard layers, organized in both prismatic and lamellar orders with up to ten-fold differences in hardness, may serve as additional protection in the case of microfailures and provide near-neutral buoyancy for the overall strong but elastic structure at the same time. These structural properties inspired different artificial and semi-artificial derivatives like foam-like solids for biomedical engineering, drug-delivery systems, additive manufacturing and other functional materials (Figure 10F,G).

The potential role of organic components in cuttlebone in the properties of composites, obtained with the use of cuttlebone in one of their components, was revealed in the comparative study of green rubbers produced by the peroxide cross-linking of natural rubber with cuttlebone (CTBO) or pure calcium carbonate (CTB) microparticles [148]. Microparticles with an average size of 29 μm were prepared by the crushing and sieving (with a 37 μm mesh sieve) of raw materials from waste stocks in the case of CTBO, while CTB microparticles with an average size of 23 μm were prepared by the calcination of CTBO microparticles at 400 °C for 30 min. Following this, 50 parts microparticles per hundred of rubber (phr) was mixed with natural rubber using a two-roll mill at 155 °C and 150 kg m^−2^ for 30 min to obtain a 1 mm thick rubber sheet. According to an atomic force microscopy (AFM) study, the rubber sheet made with CTBO showed a better interfacial adhesion between microparticles and scaffold. A simultaneous tensile/WAXD study showed the reinforcing effect of CTBO that allows the composites to better withstand stress, together with the generation of crystallites and natural rubber chain orientations. Thus, this study not only describes the procedure of the development of another type of composite, but, more importantly, reveals the role of organic components in cuttlebone, which may be exploited in another types of materials.

#### 3.6.2. Applications of Cuttlefish Bone in Biomedical Engineering

One of the most promising applications of cuttlefish bone composites is in the field of biomedical engineering; the biocompatibility and unique porous structure of cuttlefish bones make them an excellent candidate for bone regeneration scaffolds. Researchers have successfully developed composite materials by combining cuttlefish bone powder with biodegradable polymers, resulting in scaffolds that promote cell adhesion, proliferation and new tissue growth. Furthermore, the controlled release of bioactive molecules from these composites enhances the healing process, making them highly suitable for tissue engineering applications.

The structural peculiarities and outstanding rheological properties of cuttlefish bone inspired the scaffold-based guided design of new artificial materials. Thus, a chitosan/hydroxyapatite (CS/HAp) composite was proposed, in which HAp fraction was initially derived from cuttlebone using the wet precipitation method by dissolving the appropriate amounts of CaO, mixing with ammonium dihydrogen phosphate and stirring at 60 °C for 3 days; this was then aged, mixed with chitosan, frozen, treated with 1 M NaOH/ethanol at −30 °C, washed with distilled water and lyophilized. The presence of biogenic strontium (Sr^2+^), magnesium (Mg^2+^) and sodium (Na^+^) resulted in a totally non-toxic, thus environmentally friendly, and affordable, scaffold for bone regenerative medicine purposes [149].

Hydroxyapatite nanorods were developed utilizing a novel oil-bath-mediated precipitation method [150]. According to X-ray diffraction (XRD) and thermogravimetric analysis (TGA), the resulting crystallite size is about 20.86 nm and Ca/P ratio is 1.6, which is lower than human bone, and the average size is 79.05 (transversal) and 219.66 nm (longitudinal), as shown by transmission electron microscopy (TEM). These structural parameters, together with the concentration-mediated hemolytic effect and pronounced antibacterial activity, make these nanostructures promising for orthopedic applications. 

Another bioinert and strong formulation based on cuttlebone was proposed as a composite material, which also contains hydrolyzed polyvinyl alcohol (PVOH) and nano-size montmorillonite (MMT) [151]. Natural, non-purified cuttlebone particles and MMT were ground and held at 900 °C for three hours, and MMT was added to the PVOH matrix utilizing the solution casting method. The ratios were studied in the range of 1–5 phr (parts per hundred resin) of MMT and 2–5 phr of cuttlebone particles. According to rheological and X-Ray studies, 5 phr MMT provided the best reinforcing effect, and the tensile strength was maximal at 5 phr of cuttlebone particles. These ternary compositions open up great prospects for biomedical applications, yet additional studies may be proposed to further evaluate the ratios of components, since the relation between compositions and mechanical results is still not completely defined. In other words, whether the additional increasing of MMT and cuttlebone powder content may increase the target properties even more is worth studying.

#### 3.6.3. 3D Printing and Additive Manufacturing of Cuttlefish Composites

Cuttlefish bone composites have garnered interest in the field of 3D printing and additive manufacturing. The combination of the porous structure and mechanical properties of cuttlefish bones can be leveraged to create complex and lightweight structures. The incorporation of cuttlefish bone powder, or nanomaterials derived from it, into printable inks or filaments enables the fabrication of bio-inspired structures with tailored properties. Inks for 3D printing based on alginate–cuttlebone–gelatin composites were proposed as prospective bone regeneration scaffolds [152]. Formulations were prepared by the stepwise addition of ground cuttlebone powder (<300 µm) to certain compositions of sodium alginate, calcium chloride and gelatin solutions. The homogeneous thick paste-type precursor was used for printing with post-printing fixation with 1.5% calcium chloride and 1% glutaraldehyde in 1:1 H_2_O:EtOH and was thoroughly washed with distilled water. Controlled deformation studies observed with micro-CT showed that the role of alginate was crucial in preserving the mechanical stability of the obtained porous scaffolds in aqueous fluids. Biocompatibility was proven with in vitro studies of MC3T3-E1 murine pre-osteoblast adhesion and proliferation on the surface of scaffolds. The structural stability, elasticity and biocompatibility with favored mineralization make the proposed alginate–cuttlebone–gelatin composites promising for regenerative medicine [153].

#### 3.6.4. Applications in Other Functional Materials

As already mentioned, the presence of organic components in cuttlebone-derived particles may improve the structural properties of the novel materials. An ordered chamber-like macroporous silica inorganic–organic framework was obtained using the cuttlebone organic matrix and chitin using a demineralization approach [154]. Silicification (the dissolution of calcium carbonate and precipitation of silica) under base conditions led to the formation of mesoporous monoliths with silica content ranging from 65 to 75% depending on ethanol concentrations from 15 to 25%, respectively. The structure of the obtained templates may also be controlled via a partial deacetylation matrix.

Such possibilities of tuning the porosity using different physical and chemical treatments of cuttlebone scaffolds may lead to the development of functional materials with predetermined properties and functions. Thus, novel cuttlebone-derived hydroxyapatite catalyst support was proposed for TiO_2_ nanopowder [155]. The morphology and presence of titan dioxide nanoparticles on the surface of hydroxyapatite was proven by a SEM/EDX study, in correspondence with the phase composition revealed by XRD and FTIR. To study the catalytic activity, UV radiation was applied to the salicylic acid solution in the presence and absence of a catalyst. After 120 min of irradiation, up to 58% was degraded in the presence of HAp/TiO_2_ obtained by the addition of a suspension of titanium dioxide, and 61% by the sol-gel method. These values are comparable to the free suspension of TiO_2_ nanopowder, which decomposed 65% of the salicylic acid under the same conditions.

While research on cuttlefish bone composites has shown significant progress, several challenges remain. The scalability of production methods, optimization of composite properties and long-term stability of the materials are among the key areas that require further exploration. Additionally, more comprehensive studies are needed to understand the biocompatibility and potential immunological responses to cuttlefish bone composites in medical applications [156].

Cuttlefish bone composites also hold great potential in the realm of environmentally friendly materials. With increasing concerns over plastic pollution, researchers have sought alternative packaging materials that are biodegradable, sustainable and possess desirable mechanical properties [157]. By incorporating cuttlefish bone powder into biopolymers, it is possible to develop composite films and coatings with improved mechanical strength, water resistance and oxygen barrier properties. These composites show promise as eco-friendly alternatives to conventional plastic packaging materials, and also in serving as successful filler for polymers in this field, such as polyvinyl alcohol (PVA) or polyvinylpyrrolidone (PVP), for biomedical purposes (cartilage replacement) [158]. Cuttlefish bone composites can be used as adsorbents for water purification, e.g., from heavy metals [159]. The porous structure of cuttlefish bones allows for the effective removal of heavy metals and organic pollutants from water. By incorporating cuttlefish bone powder into a suitable matrix, researchers have developed composite materials that exhibit high adsorption capacity and efficiency, making them promising candidates for water treatment applications.

Recent research on cuttlefish bone composites has shed light on the exceptional properties and diverse applications of this unique biomaterial. From bone regeneration scaffolds to nanocatalysts, cuttlefish bone composites offer a compelling combination of mechanical strength, a lightweight nature and biocompatibility. While there are challenges to overcome, the continuous exploration of cuttlefish bone composites holds great promise for the development of innovative and sustainable materials in various fields.

## 4. Conclusions

The different sources of biogenic calcium carbonate, apart from their direct application in the cement production process, sustainable only in terms of reduced resource depletion, are destined to be explored for more structured, focused and added-value applications. In the present situation, they are gradually offering important outcomes in the fabrication of composites with biofiller, and as a secondary source of calcium phosphate; hence, they are entering the field of biomedical engineering, including in scaffolding and dentistry materials.

The success of the operation obviously depends on the structure of the calcium carbonate, not only the respective amounts of the various polymorphs—particularly calcite and aragonite—but also on the layers’ arrangement. Despite the fact that the calcium carbonate source is normally powdered for use, the granulometry and shape of the fragments mainly retain their original nanometric structure and this influences its physical characteristics, such as porosity and water retention, and its mechanical properties, including hardness and impact resistance, compression strength, etc.

In the case of composite filling, the matrices that are used are still prevalently oil-based, such as epoxy and polypropylene, although attempts at the application of bio-based matrices are also present in the literature. When dealing with synthetic hydroxyapatite fabrication, various methods are available and the calcination process may also be performed using different temperature and time parameters, even on the same calcium carbonate source.

To further penetrate other fields, such as biomaterials, where these biological structures can serve as a source of hydroxyapatite and, more specifically, contribute to bone tissue regeneration, biocompatibility studies are also performed. These largely assuaged concerns about contamination and relevant toxicity, although the potential for effective action may vary depending on the method used for hydroxyapatite synthesis. A big open research question concerns the interaction of bio-hydroxyapatite with biomedical metals, such as stainless steel (e.g., AISI316) and titanium alloys, such as Ti-6Al-4V, and how it differs from mineral hydroxyapatite in that aspect. Some investigations on this issue have been started and reported on.

A main unresolved question for some of the sources considered, in particular snail shells and clamshells, is the large variability among the different species. Some comparative studies do exist, although they bear limited relevance in practice so far, since the species considered for calcium carbonate sources normally refer to local markets or to consumption linked to sectors such as aquaculture. Among the various sources, eggshells represent a particularly significant sector due to their abundance in waste, together with the limited variability in the supply; eggshells have seen diffuse applications in the greatest variety of sectors. This suggests reducing its treatment in the present review with respect to the other sources, as it deserves separate consideration.

## Figures and Tables

**Table 1:** Calcium carbonate composition of most widely used shell biogenic sources.

Bio-Calcium Carbonate Source	Species	Composition	CaCO_3_ Content
*Snail shell*	*Helix pomatia* *Cornu aspersum* *Eobania vermiculata*	Normally aragonite; calcite in repaired parts Aragonite packed together by conchiolin	97% [26] 95–99% [27] Close to 100% [28]
		**Columnar** stacked aragonite	
*Chicken eggshell*	*Gallus gallus*	Calcite, yet also aragonite and vaterite	Over 95% [29]
Quail eggshell	*Coturnix coturnix*	Almost pure calcite	Around 96% [30]
*Clam shell*	*Chamelea gallina*	Calcite (10 wt.%) and aragonite (90 wt.%)	Over 99% [31]
*Mussel shell*	*Mitilus galloprovincialis*	Calcite (40 ± 4 wt%) and aragonite (60 ± 6 wt%)	Around 98.5% [32]
*Oyster shell*	*Ostrea edulis*	Almost pure calcite	Close to 100% [33]
Scallop shells	*Pecten maximus*	Almost pure calcite	98–99% [34]
*Cuttlefish bone*	*Sepia officinalis*	Pure aragonite	95% [35]

## Data Availability

No new data were created.
